# Canonical Sentence Processing and the Inferior Frontal Cortex: Is There a Connection?

**DOI:** 10.1162/nol_a_00067

**Published:** 2022-04-13

**Authors:** Nicholas Riccardi, Chris Rorden, Julius Fridriksson, Rutvik H. Desai

**Affiliations:** Department of Psychology, University of South Carolina, Columbia, SC; Institute for Mind and Brain, University of South Carolina, Columbia, SC; Department of Communication Sciences and Disorders, University of South Carolina, Columbia, SC

**Keywords:** stroke, Broca’s area, sentence comprehension, anterior temporal lobe, lesion-symptom mapping, connectivity

## Abstract

The role of left inferior frontal cortex (LIFC) in canonical sentence comprehension is controversial. Many studies have found involvement of LIFC in sentence production or complex sentence comprehension, but negative or mixed results are often found in comprehension of simple or canonical sentences. We used voxel-, region-, and connectivity-based lesion symptom mapping (VLSM, RLSM, CLSM) in left-hemisphere chronic stroke survivors to investigate canonical sentence comprehension while controlling for lexical-semantic, executive, and phonological processes. We investigated how damage and disrupted white matter connectivity of LIFC and two other language-related regions, the left anterior temporal lobe (LATL) and posterior temporal-inferior parietal area (LpT-iP), affected sentence comprehension. VLSM and RLSM revealed that LIFC damage was not associated with canonical sentence comprehension measured by a sensibility judgment task. LIFC damage was associated instead with impairments in a lexical semantic similarity judgment task with high semantic/executive demands. Damage to the LpT-iP, specifically posterior middle temporal gyrus (pMTG), predicted worse sentence comprehension after controlling for visual lexical access, semantic knowledge, and auditory-verbal short-term memory (STM), but not auditory single-word comprehension, suggesting pMTG is vital for auditory language comprehension. CLSM revealed that disruption of left-lateralized white-matter connections from LIFC to LATL and LpT-iP was associated with worse sentence comprehension, controlling for performance in tasks related to lexical access, auditory word comprehension, and auditory-verbal STM. However, the LIFC connections were accounted for by the lexical semantic similarity judgment task, which had high semantic/executive demands. This suggests that LIFC connectivity is relevant to canonical sentence comprehension when task-related semantic/executive demands are high.

## INTRODUCTION

Comprehending a spoken sentence is a complex process that requires coordination of multiple cognitive resources, such as phonological, executive, lexical, syntactic, and semantic operations. Reflecting this complexity, studies demonstrate that numerous distributed brain areas functionally contribute to sentence comprehension (Dronkers et al., 2004; Friederici, 2012; Hagoort & Indefrey, 2014; Walenski et al., 2019). Of these areas, the contribution of the left inferior frontal cortex (LIFC) in sentence comprehension remains particularly controversial. While many studies have found involvement of LIFC in sentence production and in complex sentence comprehension, negative or mixed results are often found in the comprehension of simple or canonical sentences. A more complete understanding of the role of LIFC in canonical sentence comprehension would help inform neuroanatomical models of language processing.

The LIFC has traditionally been associated with language production, but a growing number of studies report its involvement in comprehension (Desai & Riccardi, 2021; Fadiga et al., 2009; Rogalsky & Hickok, 2011). In regards to sentence processing specifically, results of neuroimaging studies of the LIFC, here defined as Brodmann Areas (BA) 44 and 45, have been inconsistent (see Kemmerer, 2021). A meta-analysis of 53 neuroimaging studies comparing sentence listening or reading to control conditions found that the inferior frontal gyrus pars opercularis (IFGoper) and pars triangularis (IFGtri) were only activated in 13 and 23 studies, respectively (Hagoort & Indefrey, 2014). Indeed, multiple neuroimaging studies have found that reading or listening to sentences passively does not activate the LIFC compared to word lists (Humphries et al., 2006; Mazoyer et al., 1993; Rogalsky & Hickok, 2009). However, an additional subanalysis within the Hagoort and Indefrey (2014) investigation revealed that semantic or syntactic demands (such as violation detection) during sentential processing reliably activated areas in the LIFC. Multiple neuroimaging studies have found LIFC activation for simple phrases and canonical word orders during tasks such as semantic/syntactic violation detection or meaningfulness judgment (Graessner et al., 2021; Schell et al., 2017; Zaccarella & Friederici, 2015).

These findings suggest that LIFC may be involved in the comprehension of simple phrases and canonical sentences, especially when there are task-related demands requiring attention to meaning or form. This predicts that damage to the LIFC should be associated with canonical/simple sentence comprehension impairments when measured by tasks that orient attention to semantic/syntactic error detection or meaningfulness. However, neuropsychological evidence supporting this prediction is relatively scarce, as briefly reviewed below. While considering these studies it is important to note that, even if noncanonical sentence comprehension is significantly more associated with LIFC disfunction than canonical comprehension, it does not follow that the LIFC does not also functionally contribute to canonical comprehension.

Two studies of patients with gliomas in the LIFC (Kinno et al., 2009, 2014) found that, compared to healthy controls, patients were impaired at two-argument active canonical sentence comprehension as measured by a sentence-picture matching task. They used voxel-based lesion-symptom mapping (VLSM) to demonstrate that these sentence impairments were significantly associated with damage to portions of the LIFC. Wilson et al. (2016) found that, compared to healthy controls, patients with primary progressive aphasia (PPA) were less accurate and responded more slowly to canonical sentences, especially for longer sentences, as measured by a sentence-picture matching task. Voxel-based morphometry (VBM) implicated LIFC and surrounding frontal cortices in the accuracy impairments. However, the VBM analysis of comprehension accuracy in that study grouped canonical and noncanonical accuracies together, and LIFC atrophy was significantly associated with slower response times for noncanonical sentences specifically, controlling for canonical performance. In a VLSM study of patients with left-hemisphere stroke, Magnusdottir et al. (2013) found that damage to white matter underlying LIFC was associated with worse sentence-picture matching performance for canonical sentences, in addition to damage to posterior temporal regions. Caramazza et al. (2005) demonstrated that, in a group of patients with Broca’s aphasia and confirmed damage to LIFC, 17 out of 38 patients were unable to perform significantly better than chance accuracy on a sentence-picture matching task of active canonical reversible sentences. However, patient performance was highly variable, and the lack of an explicit anatomical lesion-deficit association analysis makes it impossible to ascribe these impairments to LIFC damage specifically instead of other areas commonly damaged in patients diagnosed with Broca’s aphasia (e.g., insula, superior temporal gyrus (STG); Dronkers et al., 2007; Fridriksson et al., 2015).

On the other hand, many studies have found no relationship between LIFC damage and canonical sentence comprehension. In a seminal VLSM study, Dronkers et al. (2004) found no relationship between damage to IFGoper or IFGtri and sentence-picture matching for canonical sentences. Newhart et al. (2012) also found no association between LIFC damage and canonical sentences using sentence-picture matching and sentence reenactment, with LIFC damage being associated instead with auditory-verbal and working memory deficits as measured by forward and backward digit span. Also using sentence-picture matching, Rogalsky et al. (2018) found no association between LIFC damage and canonical sentence comprehension, even in a subset of patients (*N* = 11) with relatively focal lesions to LIFC. Instead, these studies find evidence either that damage to posterior temporal areas is related to worse sentence comprehension (e.g., Kristinsson et al., 2020; Thothathiri et al., 2012), or that LIFC damage is only associated with impairments for complex syntactic structures. Taken together, these studies suggest that the LIFC does not functionally contribute to canonical sentence comprehension, with its role instead being dependent on either production demands or syntactic complexity.

These mixed findings raise questions about the functional involvement of LIFC during comprehension of simple phrases and sentences. One consideration is the relative lack of task variety used in patient studies for measuring canonical comprehension. A large majority of those studies use sentence-picture matching. In that task, an *incompatible picture* trial for canonical sentences is typically created either by switching the places of the subject and object (for reversible sentences), or by replacing the subject or object with a different entity/item than what is described in the sentence (for nonreversible). Sentence-picture matching for simple sentences therefore focuses mainly on aspects of comprehension related to thematic role assignment (e.g., who is the agent and who is the patient) or single word understanding, especially related to nouns (e.g., whether the item/entity in the picture matches the word in the sentence). Other aspects of sentence comprehension, such as those related to the comprehension of verbs and their compatibility with the nouns in the sentence, are not necessarily measured. Using sentence comprehension tasks with demands that differ from sentence-picture matching may provide novel insights about the functional contribution (or lack thereof) of LIFC to canonical sentence comprehension.

Another consideration is that most patient studies of canonical sentence comprehension have used traditional lesion overlap methods, such as VLSM, to examine the relationship between behavior and brain damage. A limitation of these methods is that they can only detect areas of overlapping necrosis/gliosis. Alternatively, white matter damage/disconnection has been demonstrated to have wide-ranging consequences outside of the necrotic area and can lead to functional disruption of cortical regions that are spared by the lesion (Bonilha et al., 2014; Bonilha & Fridriksson, 2009; Bonilha, Rorden, & Fridriksson, 2014; Catani et al., 2012; Catani & ffytche, 2005; Fridriksson et al., 2007). VLSM also requires a number of patients to have overlapping damage within a given area of interest in order to detect behavioral associations, a limitation that may lead to false negatives or inconsistent results between studies depending on sample size and etiology of the brain damage (e.g., glioma, PPA, middle cerebral artery stroke). Connectivity-based lesion-symptom mapping (CLSM; Gleichgerrcht et al., 2017) can complement traditional VLSM methods by detecting effects of disrupted white matter connectivity resulting from damage anywhere along white matter tracts that connect two grey matter regions. This provides two advantages. First, it can detect effects associated with disconnection between two grey matter areas even if those areas are spared by the lesion. Second, because it is senstive to damage located anywhere along a given white matter tract (even if the specific areas of damage do not overlap between patients), it does not necessarily require the same amount of lesion overlap as VLSM. Using complementary connectivity-based measures in addition to traditional VLSM may reveal effects associated with LIFC disconnection from the larger language comprehension network that cannot be detected using VLSM in isolation.

Indeed, prior studies have raised the possibility that, despite negative VLSM findings regarding the association between LIFC damage and sentence comprehension, structural or functional disconnection of LIFC from the larger language network may have behavioral consequences (den Ouden et al., 2019; Fridriksson et al., 2018; Lukic et al., 2021; Turken & Dronkers, 2011). For example, although Dronkers et al. (2004) did not find a relationship between LIFC damage and sentence comprehension, a follow-up study in healthy participants showed that portions of the LIFC display functional and structural connectivity to the posterior temporal area that was associated with sentence comprehension in the original lesion study (Turken & Dronkers, 2011). The current investigation seeks to expand upon that finding by using CLSM to explicitly examine the behavioral associates of structural disconnection of LIFC. Additionally, Fridriksson et al. (2018) found that, while damage to LIFC was not associated with sentence comprehension impairments (canonical and noncanonical included together), white matter disconnection between the IFGoper and IFGtri did predict sentence comprehension impairments. Den Ouden et al. (2019) found similar results, but specifically for more complex syntactic structures. The current study uses similar methods, but focuses specifically on canonical sentences, to provide additional information about the role of LIFC in sentence comprehension.

Here, in a retrospective study using data collected as part of a previously existing language and cognition task battery, we used VLSM, CLSM, and region-based lesion symptom mapping (RLSM) in a group of unilateral left-hemisphere chronic stroke survivors to investigate comprehension of canonical sentences while controlling for related cognitive abilities (e.g., lexical semantics, auditory single-word comprehension, auditory-verbal short-term memory (STM)). Our focus was to investigate how damage and disrupted white matter connectivity of the LIFC and areas within two other language-related regions, the left anterior temporal lobe (LATL) and left posterior temporal-inferior parietal area (LpT-iP), affected canonical sentence comprehension and related processes. We used an auditory sentence sensibility task on declarative sentences which required participants to determine if a sentence made sense, as opposed to the commonly used sentence-picture matching task. We included four control tasks with varying lexical-semantic, executive, and phonological demands to interrogate the LIFC’s involvement in these processes (for a full description of the demands of each task, see Materials and Procedure). A visual lexical decision task with low semantic, executive, and phonological demands was used to control for lexical access. Auditory word comprehension, which has relatively higher semantic, executive, and phonological demands, was used to control for auditory single-word comprehension. A visual semantic similarity judgment task, which had high semantic and executive demands, but low phonological demands, was used to control for semantic retrieval. The forward digit span task was used to control for auditory-verbal STM.

Several hypotheses regarding the role of LIFC can be tested given this retrospective language and cognition battery. First, the *merge* hypothesis states that the LIFC is involved in the binding of two or more elements into a hierarchical structure (Zaccarella & Friederici, 2015; Zaccarella et al., 2017), starting with smaller units (e.g., *the boy*) and expanding into larger structures as the phrase or sentence continues (e.g., *the boy kicks*). This process would be important for successful completion of the sentence sensibility task but less so for the other tasks included here. Merge would be important for the sentence task because to judge the sensibility of a sentence, a hierarchical structure must be built such that the meaningfulness of the semantic and grammatical relations among the subject, verb, and object can be accepted or rejected. Two of the other tasks (visual lexical decision, visual semantic similarity judgment) have the merge operation at the most basic level (e.g., nouns and verbs are preceded by *the* and *to*, respectively), but they only contain this basic two-word structure as opposed to phrase-level combinations in the sentence sensibility task. If LIFC disruption is associated with worse sentence comprehension, but not with the other tasks, it could be taken as evidence that the LIFC functionally contributes to merge or related structure-building processes at the phrase or sentence level.

A second hypothesis is that the LIFC contributes to sentence comprehension via executive task-related demands, especially pertaining to detection of syntactic or semantic violations. Evidence for this account comes from previously discussed neuroimaging findings suggesting that LIFC is especially active in sentence comprehension tasks that require special attention to syntactic or semantic information (Hagoort & Indefrey, 2014; Hasson et al., 2006; Love et al., 2006; Rogalsky & Hickok, 2009). Because the current sentence task explicitly orients attention to the semantic meaningfulness of the sentences, support for this hypothesis would come from LIFC being a shared neural substrate for the sentence comprehension task and the semantic similarity judgment, as that task also requires explicit semantic analysis.

Third, the LIFC may contribute to sentence comprehension through auditory-verbal STM. Evidence for this hypothesis comes from neuroimaging studies showing that some areas in the LIFC are activated by both syntactic demands and STM (Matchin et al., 2017; Rogalsky & Hickok, 2011; Rogalsky et al., 2008). Neuropsychological studies also find that LIFC damage is correlated with reduced digit span and impaired comprehension of sentences (Pettigrew & Hillis, 2014). To successfully complete the auditory sentence sensibility task, participants must hold the subject, verb, and object in their STM long enough to make the meaningfulness judgment. In the current study, finding that forward digit span and sentence comprehension share the LIFC as a common neural substrate would support the auditory-verbal STM hypothesis.

Finally, the LIFC may be involved in a variety of operations that subserve general lexical-semantic processing (Fiebach et al., 2002; Heim et al., 2009; Kotz et al., 2002, 2010; Ruff et al., 2008), which may contribute to sentence comprehension. The current sentence sensibility task requires participants to understand individual words, as well as conceptual relationships between words, to judge meaningfulness. Finding that LIFC is a common neural substrate for sentence comprehension and the tasks requiring lexical-semantic search and access (i.e., auditory word comprehension, visual lexical decision, and visual semantic similarity), would support this hypothesis.

## MATERIALS AND METHODS

### Participants

Seventy-five (24 female) native English-speaking participants with unilateral left-hemisphere stroke were recruited. Fourteen participants did not complete the sentence sensibility task due to time constraints on the day of testing or technological malfunction, leaving 61 (17 female) participants for the neuroanatomical analyses (see Table 1 for demographic information). Past power analysis has demonstrated that samples of ∼50 or greater provide adequate power to detect medium to strong effects in the majority of brain areas (Kimberg et al., 2007). Participants were at least 6 months post-stroke (4.02 years ± 4.4), and a mean age at time of testing of 58.75 years ± 9.68. The Western Aphasia Battery (WAB; Kertesz, 2007) Aphasia Quotient mean score was 75.7 ± 23.6. All participants signed informed consent, and the University of South Carolina Institutional Review Board approved the research.

**Table 1.** Participant demographic information

**Participant**	**Age**	**Gender**	**Education (years)**	**Dominant hand prior to stroke**	**Aphasia type**	**WAB: AQ**
M2025	56	M	16	right	Broca’s	64.6
M2071	69	M	16	right	Broca’s	63.6
M2005	38	F	16	right	Broca’s	55.2
M2036	54	M	18	right	Broca’s	76.2
M2006	56	M	12	right	Anomic	83.2
M2002	65	M	16	right	Broca’s	80.4
M2069	77	F	18	right	Anomic	90.5
M2007	76	M	12	right	Broca’s	26.1
M2020	59	F	12	left	Anomic	86.2
M2141	79	F	16	left	None	99.1
M2142	59	M	16	right	None	99.2
M2046	56	F	14	right	Conduction	51.5
M2014	62	M	12	right	Anomic	94
M2074	55	M	16	right	Broca’s	59.4
M2061	65	M	14	right	Wernicke’s	52.7
M2078	54	M	12	left	Conduction	88.8
M2143	69	F	16	right	None	99.2
M2072	43	M	16	left	Global	23.6
M2144	65	F	12	left	None	99.6
M2145	65	M	12	right	Anomic	93.2
M2146	37	F	18	right	None	98.5
M2075	41	F	18	right	Anomic	94.2
M2059	61	M	16	right	Broca’s	58.2
M2040	52	M	16	right	Broca’s	57.5
M2076	75	M	16	right	Conduction	72.1
M2147	61	F	16	right	None	97.3
M2082	55	M	12	right	Anomic	91.1
M2079	59	M	13	right	Global	25.3
M2149	64	F	18	right	None	98.6
M2151	62	M	16	right	None	96.9
M2152	61	M	14	right	Anomic	93.1
M2153	48	M	14	right	Anomic	87.6
M2086	71	M	18	right	Conduction	73.5
M4138	67	M	14	right	Anomic	77.8
M2088	47	M	18	right	Anomic	87.5
M2044	53	F	13	right	Broca’s	74.8
M2087	69	M	16	right	Broca’s	48.9
M2155	62	F	16	right	None	98.9
M2030	62	M	16	left	Broca’s	57.2
M2156	39	F	12	right	None	96.7
M2158	51	F	18	right	None	99.6
M2159	72	M	16	right	None	99.1
M2160	49	F	16	right	None	96.6
M2094	52	M	20	right	Broca’s	64.6
M2162	59	M	13	right	None	98.4
M2031	61	M	18	right	Wernicke’s	31.2
M2103	64	M	16	right	Conduction	82.9
M4180	66	M	12	right	Conduction	45.2
M2164	68	M	16	right	None	97
M2106	60	M	16	right	Anomic	90.1
M2110	55	F	12	right	Anomic	91.3
M2109	67	F	16	right	Wernicke’s	47.8
M2114	63	M	16	right	Global	15.2
M2111	52	M	16	right	Anomic	93.4
M2029	48	M	16	right	Broca’s	43
M4209	56	M	18	right	Conduction	74.6
M2119	48	M	16	right	None	97.5
M2121	63	M	12	right	None	98.1
M2117	44	M	16	left	Broca’s	49.1
M2127	59	M	12	right	Transcortical sensory	57.8

### Materials and Procedure

#### Auditory sentence sensibility

One hundred canonical declarative and 50 low-meaningfulness or nonsense sentences were presented auditorily to the participants. Sentences were sound files recorded in a noise attenuated room by a male speaker who was instructed to read as clearly and naturally as possible, as if they were reading aloud to someone else. Sentences were denoised using Audacity audio editing software (https://www.audacityteam.org/) and ranged from 2–3.5 s in length. To preserve a more naturalistic sound for the stimuli, speech rate was not artificially modified post-recording. In a sound attenuated room, sentences were played aloud to participants on a laptop PC running E-prime software (version 1.2, Psychology Software Tools, Inc.; https://pstnet.com/products/e-prime/). Ten practice sentences were presented before the real trials to ensure that the participant could hear the sentences and understood the task. The participant was instructed to determine as quickly and accurately as possible whether the sentence made sense or not by pressing one of two response buttons. Participants had 10 s from the onset of the sentence to respond.

Fifty of the sensible sentences were literal. Half of the literal sentences described physical hand/arm actions (*The repairman bent the cable for her*), while the other half were more abstract or cognitive in nature (*The bank ignored the pleas from her*). The other 50 sensible sentences were figurative. Half of the figurative sentences were idioms (*The defense picked holes in the argument*), and half were metaphors related to physical action (*The discovery lifted this nation out of poverty*). The task was originally designed to examine literal/figurative sentence processing and action-relatedness (Fernandino et al., 2013). In the current group of patients, *t* tests revealed that response times (RT) and accuracies (ACC) did not significantly differ (RT *p* = 0.35; ACC *p* = 0.24) between the literal (*M* RT = 4,802 ms, *SD* RT = 963 ms; *M* ACC = 77.7%, *SD* ACC = 15.3%) and figurative conditions (*M* RT = 4,638 ms, *SD* = 954 ms; *M* ACC = 80.9%, *SD* ACC = 14.5%), so the two conditions were combined. There is evidence that, in sentences, high action-relatedness and figurative language may involve distributed brain areas in addition to classic language regions (e.g., Johari et al., 2021), but core language-related regions (the focus of the current study) are still expected to be involved in the majority of language processing (Binder & Desai, 2011; Binder et al., 2009), especially after collapsing all sentence types together.

Sentences followed subject-verb-object order except for four sentences that lacked an object. All verbs were transitive or ambitransitive and were used transitively except for the four aforementioned sentences. For sensible sentences, 25% of the subject nouns and 10% of the object nouns were grammatically animate. For low-sensibility sentences, 28% of the subject nouns and 4% of the object nouns were grammatically animate. Low-sensibility sentences were well formed grammatically but constructed such that the verb was incompatible with one or both of its arguments (*The taxpayer seized the planets with his small arm*; *The company twisted the shot in the dark*; *The tape rejected the air in the sky*). Detecting these sentences as nonsensible involved understanding the subject/object nouns, verb, and their semantic as well as grammatical relationships. Sensible and nonsense sentences were matched in length (number of letters, number of syllables, and number of words) and difficulty, as measured by response accuracy from a pilot study using healthy adults (Table 2; Fernandino et al., 2013). Control tasks were as follows.

**Table 2.** Means (and standard deviations) for characteristics of sensible and nonsense sentences

	**Sensible**	**Nonsense**	***t* test *p***
Letters	43.8 (7.3)	44.5 (8.9)	0.61
Syllables	11.1 (2.2)	10.6 (2.4)	0.25
Words	7.9 (1.2)	7.8 (1.7)	0.84
Accuracy in healthy adults	0.91 (0.08)	0.91 (0.08)	0.54

#### Visual lexical decision

Visual lexical decision consisted of 80 verbs, 80 nouns, and 160 phonologically plausible pseudowords. Pseudowords were chosen from the English Lexicon Project (ELP; Balota et al., 2007). Words and pseudowords were matched in number of letters, bigram frequency, orthographic neighborhood size, and visual lexical decision accuracy (Table 3). Visual lexical decision consisted of the presentation of a fixation cross (500 ms), a mask (‘########’, 100 ms), a prime (50 ms), mask (100 ms), followed by the target. The prime was the same as the target word/pseudoword in capital letters for half of the stimuli, and a consonant string also in capital letters for the other half. For the purposes of the present investigation, we do not investigate priming effects and collapse primed and unprimed trials together. Participants were instructed to indicate as quickly and as accurately as possible whether the target was a real word or not by pressing one of two buttons. Participants had 5 s to respond.

**Table 3.** Psycholinguistic variable means (and standard deviations) for words and nonwords in the visual lexical decision task

	**Words**	**Nonwords**	***t* test *p***
Length	5.53 (1.48)	5.36 (1.74)	0.51
Orthographic neighborhood	3.75 (4.85)	3.59 (3.92)	0.81
Bigram frequency	1,607.78 (713.06)	1,580.48 (738.27)	0.81
LD ACC	0.96 (0.05)	0.97 (0.03)	0.33

*Note*. LD = Lexical decision measures from the ELP database.

Half of nouns were manipulable objects (*the phone*, *the pen*), while the other half were concrete but comparatively nonmanipulable (*the ocean*, *the stadium*), as determined by body-object interaction ratings (Pexman et al., 2019). All were inanimate. Half of the verbs referred to voluntary hand/arm actions (*to pour*, *to pinch*) while the other half referred to sensory or cognitive concepts (*to observe*, *to notice*). All verbs were transitive or ambitransitive except for four (5% of verb stimuli) that were intransitive. This task was originally designed to examine priming effects, as well as effects of action-relatedness and manipulability (Desai et al., 2015). The current study focuses on core language-related regions that are expected to be involved in lexical-semantic processing for many word types (Binder & Desai, 2011; Binder et al., 2009), especially when collapsing across word categories. There were 160 trials, divided equally between words and pseudowords, in both the verb and noun versions of the visual lexical decision task. This task was the least demanding task in the battery with respect to executive and semantic processing, as it merely required participants to recognize a word as real or not, and there were no distractor items (reflected by high accuracies and low response times; Table 4). Semantic demands for lexical decision are relatively low compared to the other tasks included here, as participants do not need to explicitly access word meaning in order to successfully complete the task.

**Table 4.** Means, standard deviations (in parenthesis), and ranges for response times, accuracy, and *d*′ for all tasks

	**Auditory sentence sensibility**	**Visual semantic similarity**	**Visual lexical decision**	**Auditory word comprehension**	**Forward digit span**
RT	4,690 (920) 2,814–6,737	2,944 (616) 1,849–4,030	1,015 (327) 543–2,029	–	–
ACC	0.79 (0.14) 0.31–0.98	**0.78 (0.16) 0.45–0.98**	0.96 (0.07) 0.7–1	**0.88 (0.19) 0.13–1**	**6.6 (1.95) 0–12**
*d*′	**1.88 (1.09) −0.86–4.15**	–	**3.5 (1.1) 0.2–5**	–	–

*Note*. Bolded boxes indicate the measures used in the neuroanatomical analyses.

#### Visual semantic similarity judgment

The visual semantic similarity judgment task consisted of sets of 240 verbs and 240 nouns (see Table 5 for lexical characteristics). Each set was organized into 80 verb and 80 noun triplets such that, for each triplet, the target word was more similar in meaning to one of the two choices (e.g., ***to thrill***, *to excite*, *to harm*; bold indicates the target word). Similar to visual lexical decision, the nouns were equally divided into manipulable and concrete nonmanipulable, and the verbs into hand/arm actions and cognitive/sensory concepts. All nouns were inanimate. All verbs were transitive or ambitransitive except for two (2.5% of verbs). This task was originally designed to detect deficits related to manipulability and action-relatedness in patients (Riccardi et al., 2019, 2020). Collapsing noun and verb performance together across all categories provides a measure of general lexical-semantic processing and is expected to involve core lexical-semantic language regions. This task is also relatively executively demanding, as participants must choose the correct response in the presence of a distractor item that is from a similar semantic category, matching one of two words to the target instead of making a binary judgment. These increased lexical-semantic and executive demands are reflected by low accuracy scores and high response times compared to other control tasks (Table 4).

**Table 5.** Lexical characteristics of words in the visual lexical decision and semantic similarity judgment tasks

	**Lexical decision**	**Semantic similarity judgment**
Letters	5.32	5.40
Phonemes	3.4	4.32
Syllables	1.26	1.47
Log F	1.18	1.09
LD RT	640.75	655.5
LD ACC	0.965	0.945

*Note*. Log F = logarithmic frequency, LD = Lexical decision measures from the ELP database.

The sentence sensibility, visual lexical decision, and visual semantic similarity judgment tasks were presented on a laptop PC running E-prime software (version 1.2, Psychology Software Tools, Inc.). Participants indicated their response by pressing one of two buttons. The position of the bottom words was counterbalanced across participants. Participants could use whichever hand they preferred and were asked to respond as quickly and accurately as possible. The words remained on the screen for 5 s, after which the next triplet was presented. There were 80 trials in both the verb and noun versions.

#### Auditory word comprehension

Auditory word comprehension was administered by a licensed speech-language pathologist as part of the WAB (Kertesz, 2007). It consists of 60 real objects and pictures coming from 10 categories: real objects, drawn objects, forms, letters, numbers, colors, furniture, body parts, fingers, and right-left body parts. There are six stimuli per category. The speech-language pathologist speaks aloud the name of one of the pictures/objects, and the participant must point to the correct item. Participants are given a point for each item that they correctly point to, for a maximum of 60 points. Auditory word comprehension requires participants to comprehend a spoken word, and then to correctly recognize its corresponding visual form. Unlike the semantic similarity judgment task, it does not require explicit access to deeper semantic features in order to successfully complete the task.

#### Forward digit span

A series of digits (1 through 9) were read aloud to the participant. The participant was instructed to repeat as many of the digits as they could, in order, with series length increasing after successful repetition.

### MRI Data Acquisition

MRI data were obtained with a Siemens 3T Trio System with a 12-channel head coil and a Siemens 3T Prisma System with a 20-channel coil. Participants underwent two anatomical MRI sequences: (i) T1-weighted imaging sequence with a magnetization-prepared rapid-gradient echo (MPRAGE) turbo field echo (TFE) sequence with voxel size = 1 mm^3^, field of view (FOV) = 256 × 256 mm, 192 sagittal slices, 9° flip angle, repetition time (TR) = 2,250 ms, inversion time (TI) = 925 ms, echo time (TE) = 4.15 ms, generalized autocalibrating partial parallel acquisition (GRAPPA) = 2, and 80 reference lines; and (ii) T2-weighted MRI with a 3D sampling perfection with application optimized contrasts by using different flip angle evolutions protocol with the following parameters: voxel size = 1 mm^3^, FOV = 256 × 256 mm, 160 sagittal slices, variable flip angle, TR = 3,200 ms, TE = 212 ms, and no slice acceleration. The same slice center and angulation were used as in the T1 sequence. Diffusion tensor imaging (DTI) scans consisted of two scans with a 180° flip, TR = 4,987 ms, TE = 79.2 ms, matrix = 90 × 90, FOV = 207 × 207 mm^2^, slice thickness = 2.3 mm, and 50 transversal slices.

### Preprocessing of Structural Images

Lesions were defined in native space by a neurologist (L. Bonilha) in MRIcron (Rorden et al., 2012) on individual T2-weighted images. Preprocessing started with coregistration of the T2-weighted images to match the T-weighted images, allowing the lesions to be aligned to native T1 space. Images were warped to standard space using enantiomorphic (Nachev et al., 2008) segmentation-normalization (Ashburner & Friston, 2005) custom Matlab script (https://github.com/rordenlab/spmScripts/blob/master/nii_enat_norm.m) to warp images to an age-appropriate template image found in the Clinical Toolbox for SPM (https://www.nitrc.org/scm/?group_id=881). The normalization parameters were used to reslice the lesion into standard space using linear interpolation, with subsequent lesion maps stored at 1 × 1 × 1-mm resolution and binarized using a 50% threshold. (Because interpolation can lead to fractional probabilities, this step confirms that each voxel is categorically either lesioned or unlesioned without biasing overall lesion volume.) Normalized images were visually inspected to verify quality.

### Preprocessing of DTI Data

Diffusion data were processed in the method described in Bonilha et al. (2015). MRTrix tools were used to perform Gibbs artifacts removal (Kellner et al., 2016) and de-noising (Veraart et al., 2016). FMRIB Software Library’s (FSL) TOPUP (Andersson et al., 2003) and eddy (Andersson & Sotiropoulos, 2015) were used to attenuate spatial distortion. FSL’s dtifit was used to compute tensors, fractional anisotropy (FA), and mean diffusivity maps, and bedpost (Hernandez et al., 2013) was used to model fibers. As discussed in the previous section, the T1 scan used Statistical Parametric Mapping’s (SPM; https://www.fil.ion.ucl.ac.uk/spm/) unified normalization and segmentation. This allowed warping of atlases from standard space to the patient’s space. This was warped to native diffusion space by nonlinearly warping the T1 scan to the FA map (which has similar contrast). This allowed back-projection of our regions of interest (ROIs) into the native diffusion space. Finally, probtrackx (Hernandez-Fernandez et al., 2019) quantified connectivity. This evaluated the connectivity between each and every region in the atlas.

### DTI Connectome Creation

As described in Gleichgerrcht et al. (2017), a unique probabilistic DTI connectome was constructed for every participant using the 189 cortical regions defined by the Johns Hopkins University (JHU) atlas (Faria et al., 2012; Mori et al., 2005; Wakana et al., 2004), resulting in a 189 × 189 correlation matrix for each participant, where positive values signify greater white matter connectivity of two regions. Every participant’s probabilistic white matter map excluding the lesion was used as a mask for the estimation of their tractography. For each pair of regions, the number of streamlines arriving in one region when another region was seeded was calculated, and the connectivity was defined as the average between the number of streamlines arriving in region A when region B was seeded and the number of streamlines arriving in region B when region A was seeded. The connectivity between the regions was corrected based on the sum of the volumes of the two regions to control for larger regions inherently having a higher number of streamlines than smaller regions within the atlas. This resulted in a 189 × 189 correlation matrix of weighted connections, which was used for CLSM.

### Regions of Interest

**Figure 1.**
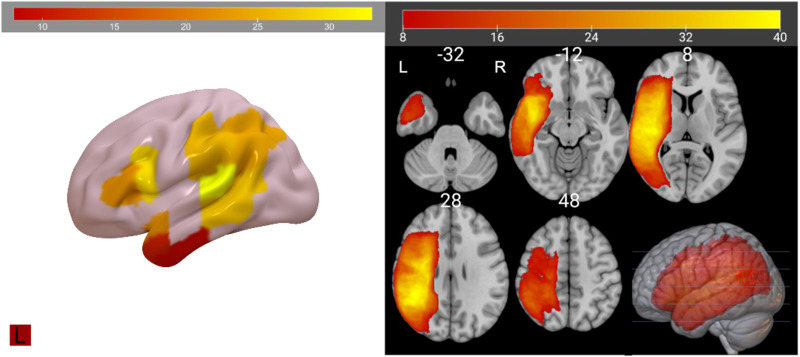
ROIs (left; min. 8, max. 33) and voxelwise lesion incidence map (min. 8, max. 40).

Nine ROIs (Figure 1), based on the JHU atlas (Faria et al., 2012; Mori et al., 2005; Wakana et al., 2004), were used for lesion-deficit analysis. We analyzed several ROIs each from LIFC, LATL, and LpT-iP. From LIFC/Broca’s area, we examined two ROIs: IFGoper and IFGtri. From LATL, we examined three ROIs: middle and superior temporal poles (MTGpole, STGpole) and the anterior portion of the inferior temporal gyrus (ITG). From LpT-iP, we examined four ROIs: posterior middle and superior temporal gyri (pMTG, pSTG), supramarginal gyrus (SMG), and angular gyrus (AG).

### Experimental Design and Statistical Analysis

#### Behavioral data

Our primary aim was to inspect the relative impairment of the sentence comprehension task (auditory sentence sensibility) factoring out the contribution of other tasks (visual lexical decision, visual semantic similarity judgment, auditory word comprehension, forward digit span). For the auditory sentence sensibility and visual lexical decision tasks, *d*′ was calculated as the difference between the *z* transforms of the proportion of hits (i.e., when a sensible sentence was identified as sensible) and the proportion of false alarms (i.e., when a nonsensible sentence was identified as sensible). A *d*′ score deviating from 0 for a participant reflects a separation between the number of correct hits and false alarms. For visual semantic similarity judgment and auditory word comprehension, the proportion out of the total possible correct was recorded. For forward digit span, the total number of digits correctly recalled, in order, was used.

#### Region- and voxel-based lesion-symptom mapping

RLSM was used within the nine ROIs (described in Regions of Interest) to identify damage related to greater impairment of the sentence comprehension task as compared to the other four tasks by regressing out performance in one condition from the other using NiiStat software (www.nitrc.org/projects/niistat/). RLSM measures the relationship between percentage of voxels damaged within an ROI and a behavioral measure. Nuisance regression used the Freedman-Lane method (Freedman & Lane, 1983), allowing for permutation-based control for family-wise error (Winkler et al., 2014). RLSM results were corrected for multiple comparisons using permutation analysis (*p* < 0.05, 1,000 permutations). Permutation analysis is a nonparametric significance test that compares a test statistic to a null distribution that is created by randomly permuting the real data (Baldo & Dronkers, 2018; Baldo et al., 2012; Kimberg et al., 2007).

Given the theoretical importance of LIFC, to increase power to detect effects VLSM was used within a restricted IFGoper and IFGtri ROI (combined into a single region for this analysis only) to investigate whether damage to voxels within this region was associated with worse sentence comprehension. VLSM binarily demarcates each voxel as either lesioned or unlesioned and tests the probability that damage to a voxel is associated with behavioral performance (Bates et al., 2003). VLSM results were thresholded at *p* < 0.001 voxel-wise and cluster-corrected to *p* < 0.05 using permutation analysis as correction for multiple comparisons (1,000 permutations). To improve power and minimize spatial bias, only voxels where at least 10% of patients had damage were considered (Baldo & Dronkers, 2018; Karnath et al., 2018). Region and voxelwise lesion incidence maps showed that we had sufficient coverage in all areas of interest (Figure 1).

#### Connectivity-based lesion symptom mapping

CLSM was used to investigate whether white matter connectivity between all regions included in the LIFC, LATL, and LpT-iP network of interest was specifically predictive of sentence comprehension impairment compared to the other tasks using nuisance regression, as described above. Left-to-left and left-to-right connections (117 in total) between the regions were considered to test for possible contributions from undamaged inter- or intra-hemispheric regions. White matter connectivity strengths were used in a general linear model to predict task performance. Alpha was set to 0.05, and significance was determined with permutation correction for multiple comparisons (1,000 permutations).

## RESULTS

### RLSM

**Figure 2.**
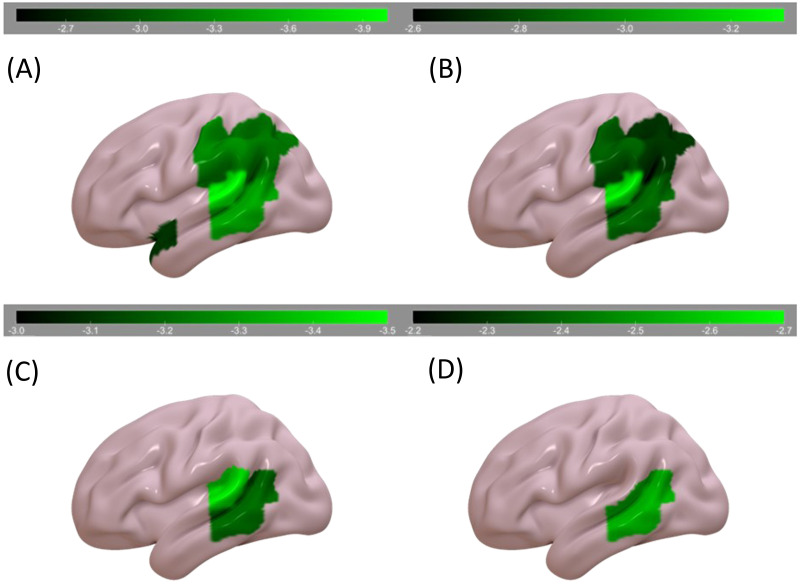
RLSM results (color bar represents *z* scores). Areas where damage was associated with: (A) worse sentence comprehension, (B) worse sentence comprehension controlling for forward digit span, (C) worse sentence comprehension controlling for visual lexical decision, and (D) worse sentence comprehension controlling for visual semantic similarity judgment.

A summary of the behavioral data can be found in Table 4. We first examined regions associated with the auditory sentence sensibility task, without including the other behavioral tasks as covariates. Auditory sentence sensibility performance was associated with percentage of voxels damaged in STGpole, SMG, AG, pSTG, and pMTG (Table 6; Figure 2). It was not significantly associated with damage in either of the LIFC ROIs.

**Table 6.** Significant RLSM regions

**Condition**	**Region**	***z* score**
**Auditory sentence sensibility**	STGpole	−2.7
	AG	−3.0
	SMG	−3.1
	pMTG	−3.4
	pSTG	−3.8
**Auditory sentence sensibility controlling for forward digit span**	AG	−2.7
	SMG	−2.9
	pMTG	−2.9
	pSTG	−3.3
**Auditory sentence sensibility controlling for visual lexical decision**	pMTG	−3.2
	pSTG	−3.5
**Auditory sentence sensibility controlling for visual semantic similarity judgment**	pMTG	−2.5

*Note*. Anatomical labels are according to the JHU atlas (Faria et al., 2012; Mori et al., 2005; Wakana et al., 2004).

Next, we individually included data from each task as a covariate, partially accounting for potential contributions of auditory-verbal STM, lexical processing, executive function, semantics, and input modality (Table 6; Figure 2). Worse auditory sentence sensibility performance, controlling for forward digit span (auditory-verbal STM), was associated with percentage of voxels damaged in the SMG, AG, pMTG, and pSTG. Controlling for visual lexical decision (lexical processing with relatively low semantic and executive demands), it was associated with percentage of voxels damaged in the pSTG and pMTG. Controlling for visual semantic similarity judgment (lexical processing with relatively high semantic and executive demands), it was associated with percentage of voxels damaged in the pMTG. No areas were significantly associated with worse auditory sentence sensibility performance when controlling for auditory word comprehension (lexical task in the auditory modality with moderate executive and semantic demands).

### VLSM

**Figure 3.**
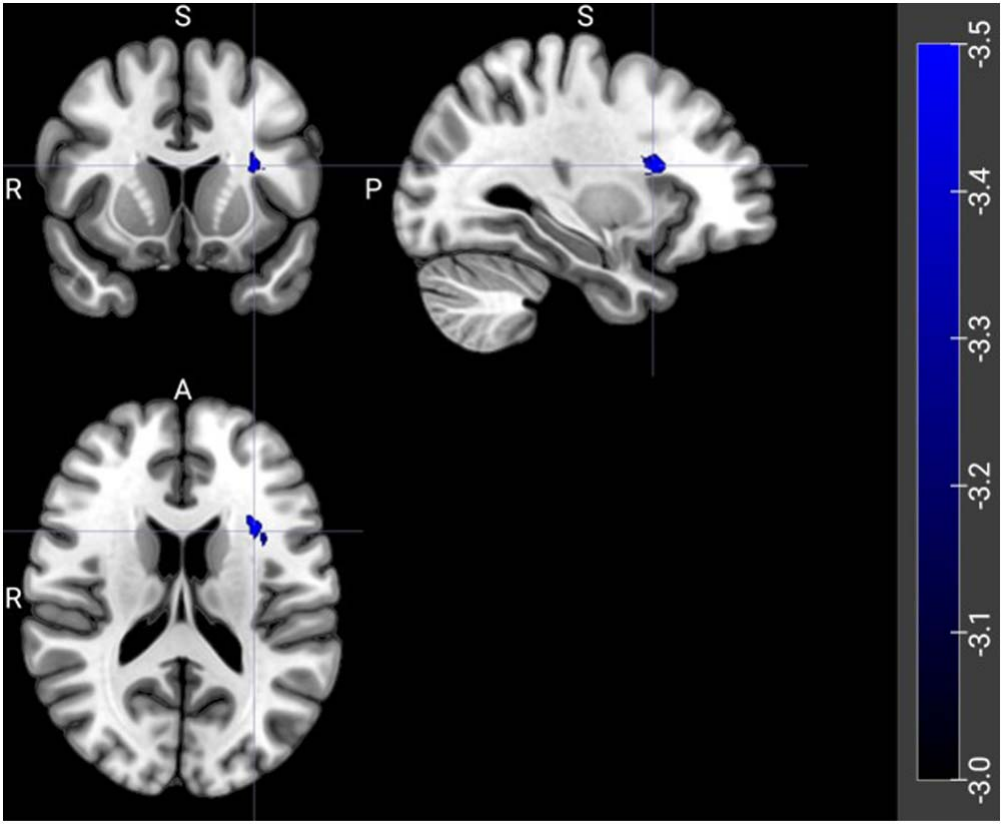
VLSM results for visual semantic similarity judgment controlling for auditory sentence sensibility. Blue voxels represent where damage is associated with worse visual semantic similarity judgment performance.

VLSM, restricted to the LIFC for increased power, showed that no voxels were associated with worse auditory sentence sensibility performance by itself, or when including any other tasks as covariates. However, worse visual semantic similarity judgment performance, controlling for auditory sentence sensibility, was associated with a cluster of voxels in the LIFC (Table 7; Figure 3).

**Table 7.** Significant VLSM peaks and Talairach coordinates

**Condition**	**Location**	**Cluster size (1 mm^3^ voxels)**	**Peak-*z* score**	**x**	**y**	**z**
**Visual semantic similarity judgment controlling for auditory sentence sensibility**	IFGtri	364	−3.8	−27	18	16

### CLSM

**Figure 4.**
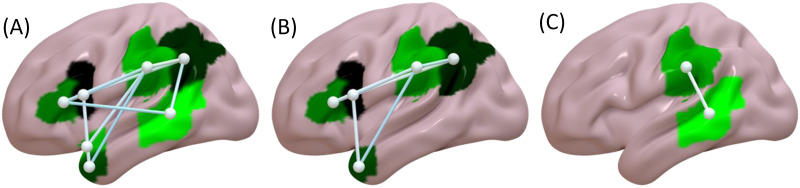
(A) White matter connections where disruption was associated with worse auditory sentence sensibility performance. (B) White matter connections where disruption was associated with worse auditory sentence sensibility performance, controlling for forward digit span, visual lexical decision, and auditory word comprehension. (C) White matter connections where disruption was associated with worse auditory sentence sensibility performance, controlling for visual semantic similarity judgment. Colors are for display only.

Worse performance in the auditory sentence sensibility task, without including the other behavioral tasks as covariates, was associated with disruption of 10 white matter connections within the left hemisphere (Figure 4A). This included connections within and between LIFC, LATL, and LpT-iP.

Worse auditory sentence sensibility performance, when including forward digit span, visual lexical decision, or auditory word comprehension as nuisance covariates, was associated with disruption of the same seven white matter connections within the left hemisphere. This included connections within and between LIFC, LATL, and LpT-iP (Figure 4B). Worse auditory sentence sensibility performance, controlling for visual semantic similarity judgment, was associated with disruption of a single white matter connection within the left hemisphere; SMG to pMTG (Figure 4C). CLSM results for all analyses are summarized in Table 8.

**Table 8.** Significant white matter connections. Disruption of these connections is associated with worse task performance.

**Condition**	**Connection**		***z* score**
**Auditory sentence sensibility**	IFGoper	SMG	3.7
		AG	3.9
		MTGpole	4.0
	IFGtri	SMG	3.8
		AG	3.8
		pMTG	3.6
	SMG	AG	4.1
		STGpole	4.0
		MTGpole	3.6
	AG	pMTG	3.8
**Auditory sentence sensibility controlling for forward digit span, visual lexical decision, and auditory word comprehension**	IFGoper	SMG	2.8
		AG	3.1
		MTGpole	3.0
	IFGtri	SMG	3.0
		AG	2.9
	SMG	AG	2.9
		MTGpole	2.7
**Auditory sentence sensibility controlling for visual semantic similarity judgment**	SMG	pMTG	2.9

## DISCUSSION

By using tasks that vary in their executive, lexical, and phonological demands, we interrogated the contributions of the LIFC, and two other language-related regions, in canonical sentence comprehension while controlling for these related cognitive processes.

### LIFC

Focusing on pars opercularis and triangularis, we examined the effects of damage to LIFC, and also of lesions affecting the white matter connectivity of LIFC within the a priori language network. RLSM and VLSM provided little evidence supporting the contribution of the LIFC to canonical sentence comprehension. Damage to LIFC was not significantly associated with worse sentence comprehension in any of the V/RLSM analyses. Results here are similar to several prior studies. For example, in the classic investigation by Dronkers et al. (2004), damage to BA 44/45 was not associated with sentence comprehension deficits for any of the several included sentence types. Similar results were reported by Newhart et al. (2012) and Rogalsky et al. (2018).

Here, we take these findings a step further by including a lexical task with relatively high semantic and executive demands, visual semantic similarity judgment. A more restrictive VLSM analysis, meant to increase power to detect effects within the LIFC, did not find a significant association between LIFC damage and sentence comprehension, even when not controlling for performance in the other tasks. However, damage to voxels within LIFC was associated with worse performance in the visual semantic similarity judgment task even after accounting for sentence comprehension performance. Visual semantic similarity judgment was one of the most semantically and executively demanding tasks included in our battery, suggesting a link between LIFC damage and impaired semantic access or control (Chiou et al., 2018; Jackson, 2021; Whitney et al., 2011).

In contrast with the V/RLSM results, CLSM demonstrated that structural connectivity of the LIFC with other regions in the a priori language network was related to sentence comprehension performance. Disruption of left-lateralized white matter connections within and between the LIFC, LATL, and LpT-iP was associated with worse sentence comprehension, not controlling for other tasks. After controlling for visual lexical decision, forward digit span, and auditory word comprehension, seven white matter connections, again consisting of links between the left LIFC, LATL, and LpT-iP, remained significantly associated with sentence comprehension. These findings demonstrate that connections between multiple left-hemisphere brain areas are important for canonical sentence comprehension, likely reflecting that successfully comprehending a sentence requires the coordination of numerous cognitive processes. Importantly, the LIFC was part of this network after controlling for auditory word comprehension, lexical access, and auditory-verbal STM. This suggests that the role of the LIFC within the sentence comprehension network goes beyond those demands. These findings do not rule out the contribution of the LIFC to these subprocesses, but they do suggest that the LIFC contributes to canonical sentence comprehension in an additional way.

Using the visual semantic similarity judgment task as a covariate allowed control for semantic and executive abilities, resulting in the absence of LIFC and LATL connections that were uniquely associated with sentence comprehension. This suggests that the LIFC and LATL connections are shared neural substrates for the sentence sensibility and semantic similarity judgment tasks. Considering this, the LIFC and LATL may contribute to canonical sentence comprehension via semantic knowledge/control and executive processes, likely associated with the task demands of the sentence sensibility task. The sentence sensibility task oriented attention to the meaningfulness of the sentences, requiring participants to identify instances where the verb and subject/object fit together to create a coherent, sensible whole. Relating to the hypotheses discussed in the Introduction, our findings closely align with the neuroimaging research suggesting that the LIFC is especially involved in sentence comprehension when the task explicitly focuses attention on semantics or is semantically demanding (Hagoort & Indefrey, 2014; Hasson et al., 2006; Love et al., 2006; Rogalsky & Hickok, 2009). In this framework, the LIFC contributes to canonical sentence comprehension via executive task-related demands, especially pertaining to detection of syntactic or semantic violations.

An alternative hypothesis partially supported by the current results is that the LIFC contributes to sentence processing via a variety of lexical-semantic operations, which are also needed by semantic similarity judgment (Fiebach et al., 2002; Heim et al., 2009; Kotz et al., 2002, 2010; Ruff et al., 2008). However, LIFC connectivity was still associated with sentence comprehension after accounting for performance in two other lexical-semantic tasks that were comparatively less demanding (visual lexical decision, auditory word comprehension), suggesting that deeper task-related semantic analysis and control may be the main factors driving LIFC connectivity involvment in the current sentence comprehension task.

The V/RLSM finding that damage to LIFC is *not* associated with canonical sentence comprehension, when contrasted with the positive CLSM finding that structural disconnection of the LIFC from the language network *is* associated with worse performance, seems paradoxical at first. One possible explanation is that the anatomy-function relationship within the LIFC has been shown to be heterogenous between individuals (Amunts et al., 1999; Fedorenko & Blank, 2020), which may impact power to detect group-level effects in fine-grained analyses such as VLSM that require overlap at the voxelwise level. However, the negative results of the RLSM anlaysis, which should not require fine-grained anatomy-function homogeneity within a region to detect effects, makes this possibility unlikely. A more likely explanation is that, as discussed in the Introduction, V/RLSM can only detect areas of overlapping necrosis/gliosis but does not consider long-range white matter tract damage that can lead to cortical disconnection and associated behavioral impairments (Gleichgerrcht et al., 2017). CLSM complements V/RLSM by being able to detect effects of disrupted white matter connectivity resulting from damage anywhere along white matter tracts that connect two grey matter regions, even if those regions are spared by the lesion. In sum, the results here build upon previous studies suggesting that disrupted LIFC connectivity has negative consequences for sentence comprehension (den Ouden et al., 2019; Fridriksson et al., 2018), and expand those findings by demonstrating that these consequences extend even to relatively simple canonical sentences and may be related to task-specific demands such as semantic analysis.

### LpT-iP

Damage to the LpT-iP, especially the pMTG, was significantly associated with worse sentence comprehension, even after controlling for performance on tasks that measured auditory-verbal STM, semantic knowledge and control, and lexical access. This aligns with the previously discussed neuropsychological evidence suggesting that the pMTG is involved in sentence comprehension, even for canonical sentences (Magnusdottir et al., 2013; Rogalsky et al., 2018). The pMTG has been identified as an important area for sentence processing, with possible contributions being mapping wordforms to their meanings (“lexical interface;” Hickok & Poeppel, 2004, 2007), processing syntax (Griffiths et al., 2013; Snijders et al., 2009), and representing semantic knowledge (Binder & Desai, 2011; Binder et al., 2009). RLSM revealed that damage to pMTG was associated with worse sentence comprehension when controlling for all tasks except for auditory single-word comprehension, a task with relatively high phonological demands. Additionally, CLSM revealed that pMTG connectivity was part of a larger network subserving sentence comprehension, but this pMTG connectivity disappeared when controlling for performance in visual lexical decision, forward digit span, or auditory word comprehension. When controlling for visual semantic similarity judgment, disruption of a single connection (pMTG to SMG) was associated with worse sentence comprehension. Considering that the pMTG did not survive any analysis using auditory word comprehension as a covariate, our results suggest that the pMTG may be a common neural substrate for auditory single-word and sentence comprehension. This aligns with the “lexical interface” hypothesis (Hickok & Poeppel, 2004, 2007), as well as studies suggesting that the pMTG may play a special role in specifically auditory language comprehension (Pillay et al., 2017). However, given the wealth of processes attributed to the pMTG, it is also possible that this area performs many different functions or that it serves as a general hub of connectivity within the language comprehension network (Turken & Dronkers, 2011).

Previous studies have demonstrated that the pSTG plays a vital role in phonological processing (Buchsbaum et al., 2001; Graves et al., 2008) and auditory-verbal STM (Leff et al., 2009; Richardson et al., 2011), both of which are related to sentence comprehension. RLSM in the current study found that pSTG damage was associated with worse sentence comprehension, but this association did not survive when controlling for auditory word comprehension performance. This supports the well-established role of pSTG in acoustic-phonological analysis (Hickok & Poeppel, 2000, 2007, 2016; Robson et al., 2012). A somewhat surprising finding was that pSTG damage was associated with sentence comprehension even after controlling for forward digit span, a measure of auditory-verbal STM. We do not interpret this as evidence against a role of pSTG in auditory-verbal STM. Instead, this finding was possibly due to forward digit span being a production task, meaning that some participants likely have displayed worse performance due to deficits in production despite relatively spared auditory-verbal STM abilities, limiting power to detect effects in brain areas related to auditory-verbal STM. Digit span is a widely used measure of auditory-verbal STM in neuropsychological studies of language comprehension (Caplan et al., 2016; Leff et al., 2009; Newhart et al., 2012; Pettigrew & Hillis, 2014; Pisoni et al., 2019; Salis, 2012), but its production component is an inherent limitation when comparing it directly to comprehension tasks. In sum, it is likely that pSTG contributes to auditory sentence comprehension via both acoustic-phonological analysis and auditory-verbal STM.

Similar to the pSTG, previous studies implicate the SMG in phonological processing, auditory-verbal STM, and the processing of lexical and sub-lexical cues in both visual and auditory modalities (Deschamps et al., 2014; Hartwigsen et al., 2010; Oberhuber et al., 2016; Sliwinska et al., 2012). Damage to SMG was associated with worse sentence comprehension, and disrupted connectivity between SMG and AG, IFG, and MTGpole predicted worse sentence comprehension after controlling for auditory-verbal STM, auditory word comprehension, and visual lexical decision performance. We interpret the SMG connectivity as reflecting the coordination of complex acoustic-phonological analysis in SMG with distributed lexical-semantic and executive processes required for successful completion of the sentence sensibility task. Further support for this interpretation was found when these connections to LATL, AG, and IFG did not survive after correcting for visual semantic similarity judgment, a task with relatively high lexical-semantic and executive demands. Instead, a single connection (SMG to pMTG) was associated with sentence comprehension. This likely reflects that the sentence comprehension task was auditory, while the semantic similarity judgment was visual, considering the previously mentioned studies indicating that SMG and pMTG play important roles in acoustic-phonological processing and auditory language comprehension, respectively.

Finally, the AG is a well-established part of the lexical-semantic system, being associated with the representation of semantic knowledge across many different tasks and concept types (Binder & Desai, 2011; Binder et al., 2009). RLSM revealed that damage to the AG was associated with worse sentence comprehension, but this did not survive after controlling for any of the lexical-semantic tasks (visual lexical decision, visual semantic similarity judgment, auditory word comprehension). CLSM showed that disconnection of the AG from IFG and SMG was associated with worse sentence comprehension after controlling for all tasks except for visual semantic similarity judgment. The RLSM findings demonstrate that the AG is a shared neural substrate for a variety of lexical-semantic tasks, while CLSM suggests that connectivity from AG to the larger language comprehension network may be shared neural correlates specifically for explicitly semantic tasks (semantic similarity and sentence sensibility judgment).

### LATL

RLSM revealed that damage to the STGpole was associated with worse sentence comprehension when not controlling for the other behavioral measures. This finding lends support to theories that implicate the LATL in sentence processing (Brennan & Pylkkanen, 2017; Brennan et al., 2012; Humphries et al., 2001, 2005). However, the fact that LATL damage was not associated with worse sentence comprehension when adding the other behavioral measures as covariates could reflect that the LATL contributes to multiple functions in addition to sentence processing, such as lexical or semantic retrieval (Lambon Ralph et al., 2017; Mesulam et al., 2013). It is also possible that regions specialized for sentence-related syntactic processing are located more inferiorly in the LATL (Humphries et al., 2005), where relatively few patients had damage, limiting power to detect effects. CLSM revealed that disconnection of the MTGpole from the IFGoper and SMG was associated with worse sentence comprehension after controlling for all tasks except for visual semantic similarity judgment. These RLSM and CLSM results, considering the already well-established role of the LATL in lexical-semantic access and retrieval (Lambon Ralph et al., 2017; Mesulam et al., 2013), suggest that the LATL contributes to canonical sentence comprehension through the representation or access of lexical-semantic knowledge.

### Limitations

This study leveraged preexisting data to interrogate the contribution of various language-related areas to canonical sentence comprehension and related cognitive processes. The tasks were not explicitly designed for the current investigation, and the retrospective nature of the study inherently limits our control over the presence of certain psycholinguistic variables. For example, while the auditory word comprehension task is a widely used clinical measure of word recognition impairments, it contains words from semantic categories that were not included in the other tasks (e.g., colors) and lacks a verb component. While our analysis focused on core language-related areas that are expected to be involved in the processing of many different types of words, it is possible that some effects observed here are associated with those lexical-semantic differences between tasks.

The current study consisted of canonical declarative sentences. Inclusion of other types of sentences, including noncanonical and more syntactically complex sentences would provide additional information about neural substrates of sentence comprehension when syntactic demands are manipulated. Additionally, it is possible that participants employed a number of strategies to aid in their performance of the sentence sensibility task, such as using lexical-semantic associations between words within the sentences, as this was not explicitly controlled for, or by using animacy clues (e.g., does the subject noun typically perform the action described by the verb). However, half of the sensible sentences were figurative (*The bank pulled the plug on the deal*). In these sentences, subject/object nouns and verbs are combined in ways that are not sensible literally, and the subject and object nouns usually have low lexical-semantic associations (e.g., *bank* and *plug*). This means that participants who heavily relied on the aforementioned strategies would still likely perform poorly overall on the task. Further, using the other lexical-semantic tasks included in the current study as covariates would also help control for this possibility.

A single meaningfulness judgment task was used. Adding other tasks, such as sentence-picture matching, could allow for direct comparisons of task type and demands. Additionally, “executive control” involves many processes, and having multiple tasks that stress control in conjunction with visual semantic similarity judgment to probe those specific processes would be helpful in understanding the specific role of the LIFC. Finally, the spatial resolution of lesion studies is inherently limited, and a fine-grained (e.g., millimeter scale) organization of function is better studied with methods such as functional magnetic resonance imaging (fMRI).

### Conclusion

VLSM and RLSM analyses suggested that the left pars opercularis and triangularis regions are not vital for canonical sentence comprehension in the context of a sensibility judgment task. LIFC damage was associated instead with impairments in a semantic similarity judgment task that had high semantic and executive demands. However, the complementary CLSM method revealed that disruption of left-lateralized white matter connections from LIFC to LATL and LpT-iP was associated with worse sentence comprehension after controlling for performance in tasks related to lexical access, auditory word comprehension, and auditory-verbal STM. Semantic similarity judgment on single words explained similar variance to sentence sensibility judgment in LIFC connectivity, consistent with the hypothesis that LIFC’s contribution to sentence comprehension is related to task-related processes when semantic demands are high. Damage to the LpT-iP, especially pMTG, predicted worse sentence comprehension after controlling for lexical access, semantic knowledge, and auditory-verbal STM, but not auditory word comprehension. This supports previous studies demonstrating that the pMTG is a vital region for sentence comprehension and suggests that the pMTG may contribute to canonical sentence comprehension by acting as an auditory “lexical interface" or general hub for auditory language comprehension.

## ACKNOWLEDGMENTS

This work was supported by NIH/NIDCD grants R01DC010783 (Rutvik H. Desai), R56DC010783 (Rutvik H. Desai), R01DC017162 (Rutvik H. Desai), and P50DC014664 (Julius Fridriksson). We thank three reviewers for their insightful comments.

## FUNDING INFORMATION

Rutvik H. Desai, National Institute on Deafness and Other Communication Disorders (https://dx.doi.org/10.13039/100000055), Award ID: R01DC010783. Rutvik H. Desai, National Institute on Deafness and Other Communication Disorders (https://dx.doi.org/10.13039/100000055), Award ID: R56DC010783. Rutvik H. Desai, National Institute on Deafness and Other Communication Disorders (https://dx.doi.org/10.13039/100000055), Award ID: R01DC017162. Julius Fridriksson, National Institute on Deafness and Other Communication Disorders (https://dx.doi.org/10.13039/100000055), Award ID: P50DC014664.

## AUTHOR CONTRIBUTIONS

**Nicholas Riccardi**: Conceptualization: Supporting; Formal analysis: Lead; Investigation: Lead; Visualization: Lead; Writing – original draft: Lead; Writing – review & editing: Equal. **Chris Rorden**: Data curation: Lead; Funding acquisition: Supporting; Methodology: Supporting; Resources: Equal; Software: Lead; Writing – review & editing: Equal. **Julius Fridriksson**: Conceptualization: Equal; Data curation: Supporting; Funding acquisition: Equal; Project administration: Lead; Resources: Lead; Supervision: Supporting; Writing – review & editing: Equal. **Rutvik H. Desai**: Conceptualization: Lead; Formal analysis: Supporting; Funding acquisition: Equal; Investigation: Equal; Project administration: Supporting; Supervision: Lead; Writing – original draft: Supporting; Writing – review & editing: Equal.
